# Visceral, general, abdominal adiposity and atherogenic index of plasma in relatively lean hemodialysis patients

**DOI:** 10.1186/s12882-018-0996-0

**Published:** 2018-08-16

**Authors:** Chaomin Zhou, Hongying Peng, Jing Yuan, Xin Lin, Yan Zha, Hui Chen

**Affiliations:** 1 Renal Division, Department of Medicine, Guizhou Provincial People’s Hospital, Guizhou Provincial Institute of Nephritic and Urinary Disease, Guiyang, 550002 Guizhou China; 2 Renal Division, Department of Medicine, Baiyun Hospital Affiliated to Guizhou Medical University, Guiyang, 550002 Guizhou China; 3 The ministry of science and education, Guizhou Provincial People’s Hospital, Guiyang, 550002 Guizhou China

**Keywords:** Visceral adiposity, Hemodialysis, Atherogenic index of plasma

## Abstract

**Background:**

Obesity is a well-established risk factor for atherosclerosis. However, it is unknown which measure of adiposity best relates to atherosclerosis in relatively lean maintenance hemodialysis (MHD) patients. We aimed to explore and compare the associations between different adiposity indices reflecting general, abdominal, visceral adiposity and arteriosclerosis risk with atherogenic index of plasma(AIP) in relatively lean MHD patients.

**Methods:**

We conducted a multicenter, cross-sectional study in Guizhou Province, Southwest China. General/abdominal adiposity indices like body mass index (BMI), waist circumference(WC), waist-height ratio(WHtR), conicity index (Ci) and visceral obesity indices including visceral adiposity index (VAI), lipid accumulation product (LAP) and the hypertriglyceridemic waist phenotype (HW phenotype) were recorded. Univariate and multivariate linear regression models were used.

**Results:**

All adiposity indices correlated positively with AIP in univariate analysis both in men and women except for Ci. After adjustment for age and traditional atherosclerosis risk factors, BMI, WC, WHtR, VAI and LAP still had associations with AIP both in men (β = 0.265, 0.153, 0.16, 0.788 and 0.74, respectively, all *P* < 0.001) and women (β = 0.34,0.199, 0.21, 0.83 and 0.74, respectively, all P < 0.001). After further adjustment for BMI, associations between AIP and VAI, LAP remained significant, but associations between WC, WHtR and AIP disappeared.

**Conclusions:**

The HW phenotype, VAI, and LAP, validated and convenient markers of visceral obesity, were superior to classical anthropometric general/ abdominal adiposity indices for atherosclerosis risk assessment, especially in relatively lean MHD patients aged 40 years or older.

## Background

Cardiovascular diseases (CVD) still represent the leading cause of mortality in maintenance hemodialysis (MHD) patients [1] and atherosclerosis is thought to be a primary contributor to CVD. It is generally acknowledged that end-stage renal disease (ESRD) patients suffer from accelerated atherosclerosis. Diffuse atherosclerosis may be observed even in early stages of chronic kidney disease (CKD) [2]. Early identification and intervention for atherosclerosis risk is therefore critical.

Adiposity is a well-known precursor of atherosclerosis. Adiposity could be widely divided into general adiposity which is usually determined by body mass index (BMI) and central adiposity with waist circumference (WC) as the most common measure. Though BMI is a confirmed indicator of obesity, fluid overload should not be overlooked, especially in MHD patients. Besides, BMI does not differentiate the types of adipose tissues as well as the body fat distribution. Though markers of central adiposity such as WC, waist height ratio (WHtR) and conicity index(Ci) provide a better estimation to evaluate proportion of body fat and fat distribution than BMI [3], they are incapable of differentiating subcutaneous fat and visceral fat, which play opposite roles in the development of atherosclerosis [4]. The importance of visceral adiposity has received increasing attention this decade [5, 6]. Many studies have indicated that it is visceral obesity rather than subcutaneous obesity that relate to metabolic abnormalities [7, 8]. Visceral adiposity index(VAI), the hypertriglyceridemic waist phenotype(HW phenotype)and lipid accumulation product (LAP) are validated and convenient markers of visceral obesity. They are reported to be associated with subclinical atherosclerosis and intracranial atherosclerotic stenosis [6, 9]. Chinese are more likely to be viscerally obesity or centrally obesity in spite of having generally low BMI [10, 11]. However, it is unknown which measure of adiposity best relates with atherosclerosis in relatively lean MHD patients.

Atherogenic index of plasma (AIP) is the logarithm of the plasma triglyceride(TG) level to high-density lipoprotein cholesterol (HDL-c) (log [TG/HDL-c])ratio [12], and is thought to be a good marker of atherogenicity and can be used in the diagnosis of subclinical atherosclerosis [13]. In the present study, we investigated and compared the sex-specific associations between different adiposity indices reflecting general adiposity(BMI), abdominal adiposity (WC, WHtR and Ci), visceral adiposity (VAI, HW phenotype, LAP) and arteriosclerosis risk with AIP in relatively lean MHD patients.

## Methods

### Participants

The Ethics Committee of The People’s Hospital of Guizhou province approved this study. This study was performed fulfilling the principles of Helsinki Declaration. We conducted a multicenter, cross-sectional study in the hemodialysis centers of 11 hospitals in Guizhou Province, Southwest China from June 1, 2016 to September 30, 2016. All MHD patients were invited to participate in our study and all participants received 4-h carbonate-based dialysis treatment twice or thrice weekly. Adult patients on standard MHD at these 11 hemodialysis centers for at least 3 months or longer were included in our study. Subjects with hearing disabilities, language barriers, mental disability, and any physical deformities, comorbid with cancer, treated with lipid-lowering drugs were excluded from our study. We also excluded subjects on any kind of nutritional support and patients with obvious edema or ascites. All participants were voluntary to take part in our study and had submitted their written consent. As BMI < 25 kg/m^2^ was considered non-obese according to ranges established for Asian populations [14], only adult patients with BMI < 25 kg/m^2^ were included in the final analysis.

### Data collection

A predesigned questionnaire was used to conduct a standardized, structured interview. The questionnaire consisted of demographic and socioeconomic information such as age, gender, personal history (coronary artery disease, hypertension, and diabetes) and details about lifestyle of the participants. Laboratory parameters including TG, cholesterol (CHOL), HDL, low-density lipoprotein-cholesterol (LDL) et al. were extracted from the medical records by researchers. Participants who did not have laboratory parameters in the past 3 months were excluded in the final analysis.

### Anthropometric measures

Anthropometric measurements were conducted in the dialysis centers after dialysis session by well-trained nurses and physicians. Weight was assessed using an electronic scale with the patients in light clothes and without shoes. Participants were asked to stand with arms hanging freely and height was acquired by a stadiometer with patient barefoot. BMI was calculated as weight (kg) divided by height squared (m^2^). WC was measured using an inelastic tape measure. Participants were asked to wear light clothes and WC was assessed midway between the last rib and iliac crest with the participants breathing out gently. Duplicate measures were taken for all measurements with a tolerance error of 1 cm for height and circumference measurements, and 1 kg for the weight measurement. A third measurement was needed if the difference of the first two measures was greater than tolerance limit. The average of the two closest measurements was recorded at last. WHtR was calculated according to the following formula: WC (cm) /height (cm) [15]. CI was calculated according to the formula [16, 17]: WC (m) / [0.109 × square root of weight (kg) /height (m)]. LAP index was calculated according to the published formula [18]: (WC (cm)-65) × TG (mmol/l) for men and (WC (cm) -58) × TG (mmol/l) for women.

### Definition of VAI score

The VAI score was derived according to a published formula [19]:

$$ \mathrm{Males}:\mathrm{VAI}=\left[\mathrm{WC}/39.68+\left(1.88\times \mathrm{BMI}\right)\right]\times \left(\mathrm{TG}/1.03\right)\times \left(1.31/\mathrm{HDL}\right) $$

$$ \mathrm{Females}:\mathrm{VAI}=\left[\mathrm{WC}/36.58+\left(1.89\times \mathrm{BMI}\right)\ \right]\times \left(\mathrm{TG}/0.81\right)\times \left(1.52/\mathrm{HDL}\right)\ \left(\mathrm{both}\ \mathrm{TG}\ \mathrm{and}\ \mathrm{HDL}\ \mathrm{were}\ \mathrm{in}\ \mathrm{mmol}/\mathrm{l}\right) $$

### Definition of the HW phenotype

The HW phenotype was defined as elevated WC (> 90 cm in men and > 80 cm in women), along with an elevated plasma TG concentration ≥ 1.7 mmol/l for both genders [20]. As the HW phenotype was proposed to be a surrogate marker of visceral obesity, subjects were divided into 3 groups by the presence of the HW components. Group 1, normal WC (WC < 90 cm in men or < 80 cm in women) and normal TG level (TG < 1.7 mmol/L) for both genders; Group 2, solely WC increase (WC ≥ 90 cm in men or ≥ 80 cm in women, along with TG < 1.7 mmol/l) or solely TG increase (WC < 90 cm in men or < 80 cm in women along with TG ≥ 1.7 mmol/l); Group 3, increased WC and TG level (WC ≥ 90 cm in men or ≥ 80 cm in women, and TG ≥ 1.7 mmol/l).

### Statistical analysis

Statistical analyses were conducted using SPSS software (version 13.0 for windows; SPSS, Chicago, IL, USA). Continuous variables were presented as median and interquartile range due to their skewed distribution. Mann–Whitney U test was used to compare continuous variables in male and female participants. Categorical variables were expressed as percentages. The chi-squared test was used to assess differences in proportions between groups for categorical variables. Spearman correlation analysis was used to evaluate the association between the HW phenotype and AIP. Univariate and multivariate linear regression models were used to examine the associations between various markers of obesity (BMI, WC, WHtR, Ci, VAI and LAP) and AIP. A two-tailed *P* value less than 0.05 was considered statistically significant.

## Results

Initially there were 939 participants included in our study, 30 participants were excluded from our final analysis because of missing data for TG, CHOL, HDL and LDL. The main anthropometric and clinical characteristics of the study population were presented in Table 1. As shown in Table 1, levels of TG, HDL, LDL and CHOL in women were higher as compared to men. Men exhibited increased BMI and WC than women (*P* < 0.05). There was no statistical difference in AIP between male and female participants. More than 80% of the participants were aged 40 years and older. 21.7% women had the HW phenotype, while only 8.8% men had this phenotype. VAI and LAP were higher in women as compared to men (*P* < 0.001).

**Table 1:** Characteristics of the study population stratified by sex

Characteristic	Men (*n* = 551)	Women (*n* = 358)	*P* value
Age (years)	56(43,67)	57(44,70)	NS
Age ≥ 40 years(%)	80.5	80.1	NS
History of smoking (%)	55.5	6.1	< *0.001*
History of high blood pressure (%)	83.2	80.6	NS
History of diabetes N(%)	199(36%)	113(31%)	NS
Waist circumference (cm)	82.7(76.25,88.6)	79 (73.7,85.13)	< *0.001*
Body mass index	21.45(19.59,23)	20.81(18.94,22.65)	< *0.001*
Waist-to-Height Ratio	0.50 (0.46,0.54)	0.51(0.47,0.55)	< *0.001*
Conicity index	1.27(1.21,1.33)	1.29(1.21,1.35)	NS
Visceral adiposity index	1.56(0.94,2.61)	2.32(1.43,3.72)	< *0.001*
Lipid accumulation product	21.28(12,37.34)	31.92(17.6,52.5)	< *0.001*
Atherogenic index of plasma	0.07 (−0.14,0.29)	0.07(− 0.13,0.29)	NS
Hypertriglyceridemic waist, %	8.8	21.7	< *0.001*
Triglycerides (mmol/L)	1.27(0.93,1.86)	1.46(1.09,2.2)	< *0.001*
High density lipoprotein (mmol/L)	1.08(0.88,1.3)	1.25(1.01,1.52)	< *0.001*
Low density lipoprotein (mmol/L)	2.17(1.7,2.72)	2.34(1.78,3.01)	*0.001*
Total cholesterol (mmol/L)	3.78(3.17,4.35)	4.24(3.65,5.03)	< *0.001*

Data are presented as n (%) or median and interquartile range. NS, non- significant (P > 0.05)

### Relationship between various measures of general/central obesity and AIP

**Fig. 1:**
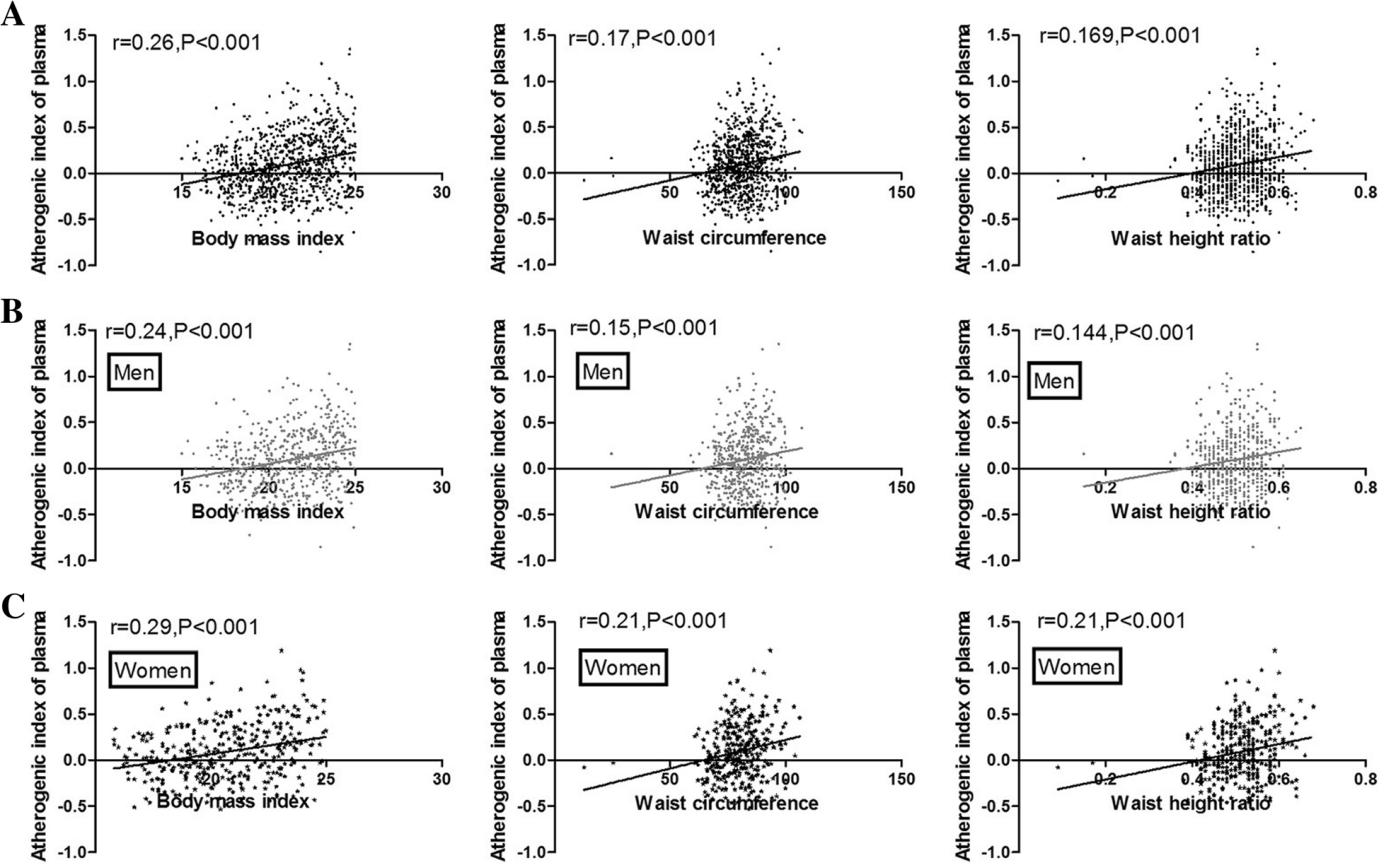
Relationship between body mass index (BMI), waist circumference(WC), waist-height ratio(WHtR) and atherogenic index of plasma (AIP). **a** entire subjects; **b** Men; **c** women

Univariate correlations between various measures of general/central obesity and AIP were shown in Fig. 1. Ci had no significant correlation with AIP(*P* > 0.05). BMI, WC and WHtR were positively associated with AIP in the entire population (*r* = 0.26, 0.17, 0.169, respectively, all *P* < 0.001). As shown in Fig. 1, BMI showed stronger correlation with AIP (*r* = 0.24 for men, *r* = 0.29 for women, P < 0.001) than WC (*r* = 0.15 for men, *r* = 0.21 for women, P < 0.001) and WHtR (*r* = 0.144 for men and r = 0.21 for women, P < 0.001) in both men and women. As shown in Table 2, BMI, WC, WHtR still correlated with AIP after adjustment for age and traditional atherosclerosis risk factors such as history of smoking, history of high blood pressure and history of diabetes both in men and women. However, the associations disappeared after further adjustment for BMI(*P* > 0.05).

**Table 2:** Comparison of the association of various measures of adiposity with AIP after adjusting for traditional atherosclerosis risk factors and BMI

	Model 1		Model 2	
	β	P value	β	P value
Men				
BMI	0.265	< 0.001		
WC	0.153	0.001	*NS*	*NS*
WHtR	0.16	0.001	*NS*	*NS*
VAI	0.788	< 0.001	0.77	< 0.001
LAP	0.74	< 0.001	0.725	< 0.001
Women				
BMI	0.34	< 0.001		
WC	0.199	0.001	*NS*	
WHtR	0.21	< 0.001	*NS*	
VAI	0.83	< 0.001	0.816	< 0.001
LAP	0.74	< 0.001	0.73	< 0.001

Model 1: adjusted for age, history of smoking,history of high blood pressure and history of diabetes;

Model 2: adjusted for above + BMI

BMI, body mass index; WC, waist circumference; WHtR, waist height ratio; VAI, visceral adiposity index; LAP, lipid accumulation product

NS, non- significant (P > 0.05)

### Relationship between markers of visceral obesity and AIP

As shown in Table 3, there was a moderate correlation between HW phenotype and AIP (*r* = 0.601 for men, *r* = 0.693 for women, *p* < 0.0001). VAI and LAP were significantly and positively correlated with AIP (r_VAI_ = 0.802, r_LAP_ = 0.71for men, r_VAI_ = 0.84, r_LAP_ = 0.72 for women, p < 0.0001). VAI showed the strongest correlation with AIP (β = 0.8, *P* < 0.001) in overall subjects. After adjustment for age and traditional atherosclerosis risk factors (i.e. history of smoking,history of high blood pressure and history of diabetes), VAI and LAP still had strong associations with AIP in men (β = 0.788 and 0.74, respectively, both P < 0.001) and women (β = 0.83 and 0.74, respectively, both P < 0.001) as shown in Table 3. These associations remained, although slightly attenuated, after further adjustment for BMI (all P < 0.001).

**Table 3:** Pearson / Spearman correlation coefficients for visceral obesity markers and AIP

	Visceral adiposity index	Hypertriglyceridemic waist phenotype	Lipid accumulation product
Overall (*n* = 909)			
Standardized coefficients β	0.8	0.644	0.70
P	< *0.001*	< *0.001*	< *0.001*
Male (n = 551)			
Standardized coefficients β	0.802	0.601	0.71
P	< *0.001*	< *0.001*	<* 0.001*
Female (n = 358)			
Standardized coefficients β	0.84	0.693	0.72
P	< *0.001*	< *0.001*	< *0.001*

## Discussion

Adiposity is a known major determinant of atherosclerosis in the general population. Whether adiposity indexes are associated with atherosclerosis in relatively lean MHD patients is unknown. Moreover, there are few studies to compare the relative strength of the associations between obesity indexes reflecting general, abdominal, visceral adiposity and atherosclerosis risk. To the best of our knowledge, this is the first study investigating and comparing associations between general, abdominal and visceral adiposity, estimated by obesity indexes and atherosclerosis risk with AIP in relatively lean MHD patients. We found that increase of anthropometric obesity indicators, especially indicators reflecting visceral adiposity was positively associated with atherosclerosis risk even in relatively lean MHD patients. Surrogate markers of visceral obesity were superior to traditional anthropometric obesity indicators, reflecting general and abdominal obesity in identifying atherosclerosis risk. Moreover, associations between markers of visceral obesity and AIP were independent of BMI and traditional atherosclerosis risk factors in relatively lean MHD patients.

The AIP has been suggested to be a good marker of atherogenicity [13]. It was found to be associated with high cardiovascular risk and adverse clinical outcomes [12, 21]. In the present study, BMI showed the strongest correlation with AIP in entire population among various measures of general/central obesity (BMI, WC, WHtR and Ci). While Brodsky demonstrated an inverse relationship between obesity, generally determined by BMI, and subsequent aortic atherosclerosis, which has been termed the “obesity paradox” [22]. In fact, “obesity paradox” exists in diverse diseases including ESRD [23]. “Obesity Paradox” is still controversial [24] and several studies propose that the use of BMI, which is a poor indicator of body fat distribution, as a determinant of obesity may be part of the explanation for “Obesity Paradox” [25]. In addition, fluid overload should be considered especially in ESRD patients. Central obesity is thought to be more pathogenic and more important as a predictor of cardiovascular metabolic disease compared to general obesity [26, 27]. General obesity as assessed by BMI, however, showed a stronger relation with AIP than central obesity as assessed by WC, WHtR in our study. Antoninus et al. [28] demonstrated that a significant correlation was obtained between each of the anthropometric measures and AIP in non-obese sedentary men except for Ci. Consistent with the findings in Antoninus’s study, Ci showed no association with AIP in our study. In their study, BMI had a stronger correlation with AIP compared with other obesity indicators such as WC, WHtR, WHR, which was similar to our results. Though WC, WHtR correlated with AIP, the associations were weak and depend on BMI in our study. Actually, these results were in compliance with previous studies which suggested that BMI was a more powerful predictor of body fat percent than WC and Ci [29] and BMI was a stronger predictor of metabolic syndrome (MetS) than WC and Ci [30]. As we all know MetS showed significant relationship with carotid atherosclerosis [31].

Though central obesity indicators such as WC and WHtR were recommended to be relatively ideal markers of obesity [32]. It is rather difficult for these markers to identify subcutaneous and visceral adiposity. The importance of visceral obesity has been emphasized by a lot of studies [5, 6]. Studies have indicated that males and postmenopausal females tend to deposit more visceral fat [33]. More than 80% patients were above 40 years in the present study. Most women reach menopause during age 45 to 55 years, but it may occur from age 40 to 60 years [34]. We could speculate therefore, most of our female patients were menopausal. This might be a reasonable explanation for women were more likely to have visceral obesity in the present study, as more women had the HW phenotype and LAP, VAI were much higher in women compared to men. Our previous studies and other studies from the Chinese population had come to similar conclusions [20, 35, 36]. We found strong positive associations between visceral obesity indicators (VAI, LAP and HW phenotype) and atherosclerosis risk both in men and women. VAI and LAP seemed to have a stronger relation with AIP in women compared to men. These associations were independent of BMI and traditional atherosclerosis risk factors such as age, history of smoking,history of high blood pressure and history of diabetes. Our results might partially contribute to the middle-aged population as more than 80% subjects were aged 40 or older, and this group was particularly exposed to increased risk of developing the cardiometabolic profile. Our results were supported by previous studies which suggested that TG/HDL-C concentration was highly correlated with VAI [19, 37]. Another study has reached a similar conclusion [38]. A recent study has suggested that visceral fat mass, estimated by Dual-energy X-ray absorptiometry (DXA) was negatively associated with HDL cholesterol and positively associated with TG [39]. As we know AIP is logarithmic ratio of TG/HDL-C. Data also suggest that VAI is positively correlated with coronary artery calcium score (CACS), a marker of subclinical atherosclerosis [40] and the association is still significant even after adjusting for confounding variables, including BMI. Previous studies have indicated that the HW phenotype is highly associated with CACS [41] and LAP outperforms BMI in identifying metabolic risk factors [42] and predicting all-cause mortality [43]. A previous study suggested that accumulated abdominal visceral fat was associated with artery intima-media thickness [44].

Exact mechanisms related to the associations between markers of visceral obesity and atherosclerosis risk in relatively lean MHD patients are yet to be clearly established. Data suggest that visceral fat depots and hypertrophic adipocytes drive the secretion of proinflammatory cytokines such as interleukine-6 (IL-6), tumor necrosis factor-alpha(TNF-a). Many of these cytokines hav**e** a detrimental effect on atherogenesis, plaque progression and thrombosis [45, 46]. Animal experiments demonstrate that enhanced atherosclerosis can be attributed to a cross-talk between visceral fat and the vasculature, which is mediated by the release of proinflammatory cytokines [47]. But these explanations have not been precisely defined in human and are likely to be multifactorial, further studies are needed.

Limitations in the current study deserve to be illustrated. First, the cross-sectional nature of our study disabled us to make causal inferences between obesity and atherosclerosis in non-obese MHD patients, prospective studies are needed. Second, subjects in this study were all Chinese, extrapolating results to other populations should be interpreted cautiously. Third, there was an age imbalance in our study population, over 80% participants in our study were aged 40 years and older. Fourth, the sample size was relatively small. However, this is the first study to investigate and compare the sex-specific associations between different adiposity indices reflecting general adiposity, abdominal adiposity, visceral adiposity and arteriosclerosis risk with AIP in relatively lean MHD patients. Therefore, despite these limitations, our study is valuable.

## Conclusions

The HW phenotype, VAI, and LAP, validated and convenient markers of visceral obesity, were superior to classical anthropometric general/ abdominal adiposity indices for atherosclerosis risk assessment, especially in relatively lean MHD patients aged 40 years or older.

## Abbreviations

AIP: Atherogenic index of plasma
BMI: Body mass index
Ci: Conicity index
HW phenotype: Hypertriglyceridemic waist phenotype
LAP: Lipid accumulationproduct
MHD: Maintenance hemodialysis
VAI: Visceral adiposity index
WC: Waist circumference
WHtR: Waist-to-height ratio
